# A data-driven model to study utero-ovarian blood flow physiology during pregnancy

**DOI:** 10.1007/s10237-019-01135-3

**Published:** 2019-03-05

**Authors:** Jason Carson, Michael Lewis, Dareyoush Rassi, Raoul Van Loon

**Affiliations:** 1 College of Engineering, Swansea University, Bay Campus, Fabian Way, Swansea, SA1 8EN UK; 2 College of Human and Health Sciences, Swansea University, Singleton Campus, Singleton Park, Swansea, SA2 8PP UK

**Keywords:** Pregnancy, 1D–0D cardiovascular network, Physiological adaptation, Data-driven modelling, Utero-ovarian flow

## Abstract

In this paper, we describe a mathematical model of the cardiovascular system in human pregnancy. An automated, closed-loop 1D–0D modelling framework was developed, and we demonstrate its efficacy in (1) reproducing measured multi-variate cardiovascular variables (pulse pressure, total peripheral resistance and cardiac output) and (2) providing automated estimates of variables that have not been measured (uterine arterial and venous blood flow, pulse wave velocity, pulsatility index). This is the first model capable of estimating volumetric blood flow to the uterus via the utero-ovarian communicating arteries. It is also the first model capable of capturing wave propagation phenomena in the utero-ovarian circulation, which are important for the accurate estimation of arterial stiffness in contemporary obstetric practice. The model will provide a basis for future studies aiming to elucidate the physiological mechanisms underlying the dynamic properties (changing shapes) of vascular flow waveforms that are observed with advancing gestation. This in turn will facilitate the development of methods for the earlier detection of pathologies that have an influence on vascular structure and behaviour.

**Electronic supplementary material:**

The online version of this article (10.1007/s10237-019-01135-3) contains supplementary material, which is available to authorised users.

## Introduction

The human cardiovascular system undergoes significant physiological and structural adaptations during healthy pregnancy. Cardiac output increases by 30–50% (Meah et al. 2016; Soma-Pillay et al. 2016) as a result of increases in both stroke volume and heart rate, blood volume increases by up to 40%, vascular compliance is increased, and total peripheral resistance is reduced. These changes maintain a relatively uniform mean arterial blood pressure throughout pregnancy, with blood pressure decreasing slightly from the first to second trimester and then increasing towards term. Pathologies can result if appropriate cardiovascular adaptation does not occur (for example, hypertension may be caused by inadequate reduction in peripheral resistance relative to increased cardiac output; hypotension may result if blood volume does not increase sufficiently relative to the extent of vascular remodelling). More clinically significant pathologies such as placental insufficiency [which can lead to reduced foetal growth (Gagnon 2003; Neerhof and Thaete 2008; Krishna and Bhalerao 2011)] and pre-eclampsia (hypertension with proteinuria, one of the most common complications of pregnancy) are often associated with significantly elevated placenta (uterine) vascular resistance (Thornton et al. 2010; Payne et al. 2011; Ayoubi 2011; Gathiram and Moodley 2016).

The maternal cardiovascular system also undergoes several major physical adaptations. These include remodelling of the heart and blood vessels, which cause changes in blood flow distribution and flow rates. Heart remodelling (Hall et al. 2011) results in increased wall thickness and increased left ventricular mass (Haas et al. 2017). The creation and development of the placenta (a specialised organ that facilitates nutrient exchange between maternal and foetal systems) represent a significant change compared with non-pregnant cardiovascular structure. Vascular network remodelling results in increased vascular lumen diameters and increased vessel compliance, and causes notable changes to the micro-structure of the spiral arteries in the utero-ovarian system (Burton et al. 2009; Cartwright et al. 2010; Harris 2010; Mandala and Osol 2011; Pijnenborg et al. 2006; Whitley and Cartwright 2009).

The most significant regional changes (increases) to blood flow are those supplying the uterus, ovaries, kidneys and skin. In particular, flow is increased by approximately 1500% to the uterine artery (which supplies approximately 90% of the blood to the uterus in non-pregnant individuals) and to the utero-ovarian communicating artery (which supplies approximately 10% of the blood to the uterus in non-pregnant individuals). During pregnancy, the utero-ovarian communicating artery may increase in diameter sufficiently to supply the uterus with the majority of its blood supply needs (Burbank 2009).

None of the currently available models of the maternal circulation consider the various pathways of blood supply to the uterus, generally only considering the uterine arteries. To date, cardiovascular modelling in pregnancy has largely focused on representing the foetal system (Pennati et al. 1997; Garcia-Canadilla et al. 2014), the placenta (Chernyavsky et al. 2010) and modelling blood flow in the umbilical cord (Queyam et al. 2017; Shah et al. 2017). A model of the feto-maternal circulation was presented in Ménigault et al. (1998); however, the model mainly considered the foetal circulation, with the placenta and the uterine arteries being the only maternal vasculature included in the model. A lumped model of the maternal system has been recently proposed (Corsini et al. 2017); however, blood flow to the uterus was considered only via one pathway. Blood vessels in the utero-ovarian system form a loop, such that the uterus can be supplied from the ovarian arteries (which originate from the descending aorta) and from the uterine arteries (which originate from the internal iliac arteries), although this loop has not been included in previous models. The clinical detection of many vascular pathologies in pregnancy requires estimated measures of arterial stiffness, which are dependant on wave propagation and wave reflection phenomena. The most common methodology used to capture wave propagation phenomena is the distributed 1D model, which often use lumped models to represent the micro-circulation (Sherwin et al. 2003; Bessems et al. 2007; Alastruey et al. 2008a, b, 2009), which is the type of model presented in this paper. The wave propagation behaviour of these 1D models has been validated using in vivo (Reymond et al. 2011; Mynard et al. 2014), in vitro (Matthys et al. 2007; Bessems et al. 2008; Alastruey et al. 2011; Saito et al. 2011) measurements. Furthermore, the 1D model solutions compare favourably with 3D models, with several in silico studies being performed (Grinberg et al. 2010; Xiao et al. 2013). However, one of the main advantages the 1D methodology has over the 3D models is that the computational cost is much lower. Due to the large computational cost of 3D models, it would be unrealistic, if not impossible to implement a 3D computational fluid dynamics approach for a large network such as the one proposed. Generally only small networks are investigated using 3D modelling, such as the coronary circulation or cerebral circulation. Furthermore, as pregnancy adaptation heavily relies on compliance changes in the uterine region, an assumption of a rigid wall would not be reasonable; hence, the more computationally expensive 3D fluid–structure interaction modelling approach would have the be performed, which would require a lot more input data. Thus, for this problem the 1D modelling approach is preferred.

Notably, uterine artery blood flow has been studied extensively using Doppler ultrasound to measure the shape of the uterine flow waveforms (Dickey 1997; Gómez et al. 2008; Ferrazzi et al. 2011; Salavati et al. 2016; Khong et al. 2015; Sciscione and Hayes 2009; Barati et al. 2014; Giordano et al. 2010; Lin et al. 1995; Schulman et al. 1986; Gómez et al. 2005; Papageorghiou et al. 2005; Campbell et al. 1987; McKelvey et al. 2017; Bower et al. 1993; Chan et al. 1995; Matijevic and Johnston 1999; Papageorghiou et al. 2004; Antsaklis et al. 2000; Chien et al. 2000; Axt-Fliedner et al. 2004; Harrington et al. 2003; Frusca et al. 1998). Aberrant flow waveform shapes (and thus flow rates) have been associated with detriment to pregnancy outcome (Dickey 1997; Gómez et al. 2008) and restricted foetal growth (Ferrazzi et al. 2011). However, previous attempts to measure flow rates from the utero-ovarian communicating artery have been unsuccessful (Pates et al. 2010; Browne et al. 2015) owing to the complex anatomy and flow behaviour in the pelvic region. Hence, it would be useful to estimate the flow through the utero-ovarian communicating artery using a model.

In this paper, a comprehensive 1D–0D closed-loop multi-scale model of the maternal circulation is presented. The model uses physiological measurements collected from various studies (Carpenter et al. 2015a, b, c, 2016a, b; D’Silva et al. 2013) to tune and validate model performance. Simulations are performed using data from two pregnant women during the first, second and third trimesters, and for the same individuals' post-partum. We demonstrate that this model can provide realistic estimates of utero-ovarian wave propagation phenomena and of the volume of blood supplied to the uterus from the utero-ovarian communicating artery.

## Methods and materials

The validated closed-loop model of Mynard and Smolich (2015) is used as the basis for the development of the female-specific, closed-loop model presented in this work. The main addition is the inclusion of the vessels and vascular beds which supply the female reproductive system. An initial and iterative parameter estimation technique is presented and tested using the in vivo measurement data of two participants at several stages during pregnancy and post-pregnancy.

### Physical domain

#### 1D vessel network

**Fig. 1:**
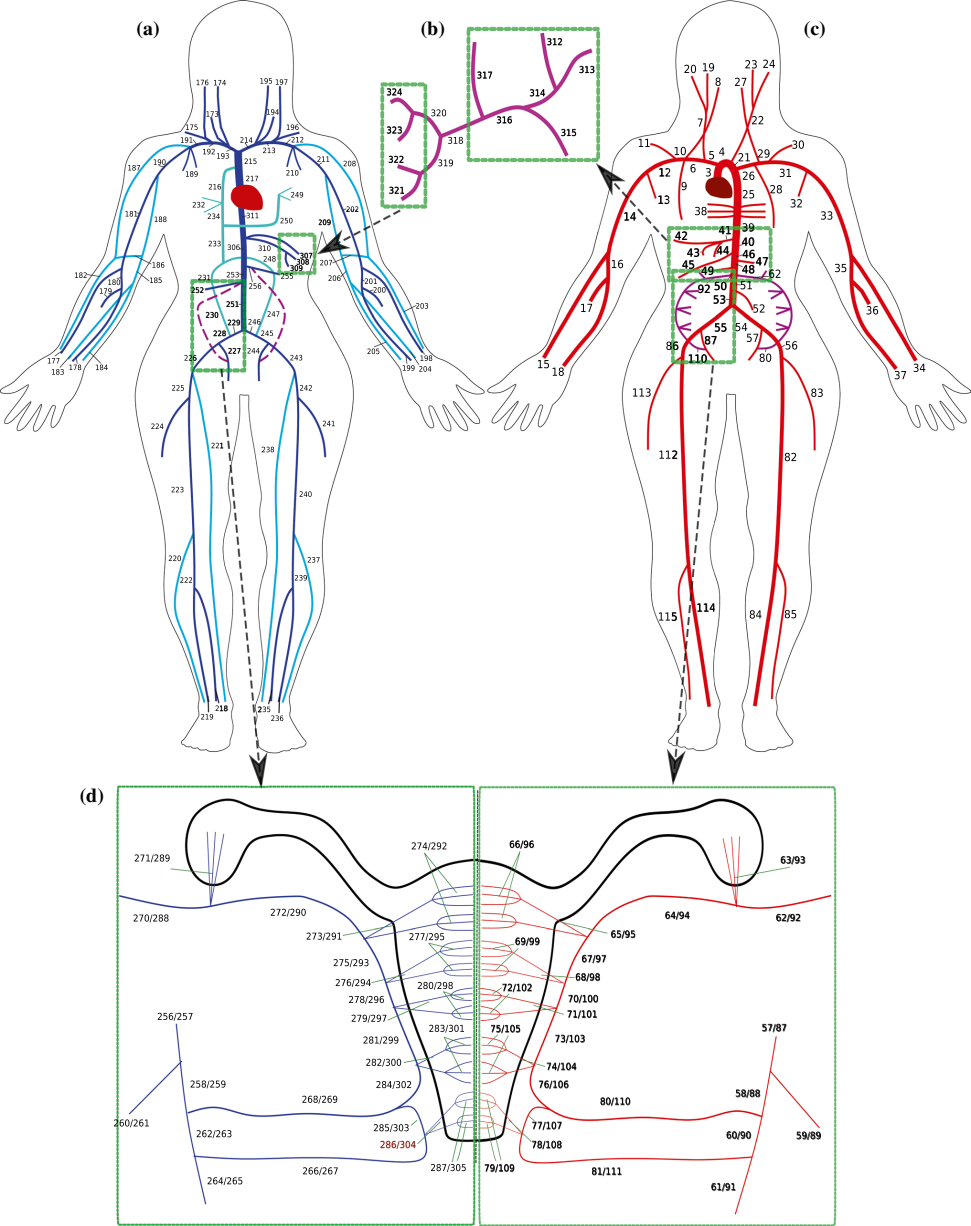
Overview of the main 1D network. **a** Systemic veins, **b** hepatic portal veins, **c** systemic arteries, **d** utero-ovarian vessels. Vessel numbers correspond with the network given in the supplementary material

The 1D network that represents the utero-ovarian circulation is described in Sect. 2.1.2. In the systemic venous system the following vessels are also added: the ascending lumbar veins, azygos vein, hemiazygos and accessory hemiazygos veins. These vessels provide an important alternative pathway for blood to travel from the lower body to the heart, which could be important in cases of supine hypotensive syndrome, where the foetus can cause the inferior vena cava to partially collapse, lowering venous return. A supplementary file contains information on the full network, which consists of 513 1D vessels and 62 vascular beds. The majority of 1D vessel lengths and diameters used in this paper are from Mynard and Smolich (2015) and are from either published studies or anatomical considerations. Any vessels for which data in the literature cannot be found utilises a variation in Murray’s law (Murray 1926) with an exponent of 2.76. The anatomical configurations for the systemic arteries and veins are shown in Fig. 1. The remainder of the model network (coronary and pulmonary, arteries and veins) is identical to that of Mynard and Smolich (2015).

#### Utero-ovarian circulation

The utero-ovarian system undergoes the most substantial structural adaptation during pregnancy. This includes an increase in unstressed vessel area and compliance (via the trophoblast invasion of maternal cells). Moreover, an entirely new organ is formed and developed over the course of pregnancy, i.e. the placenta. The placenta is an organ of low resistance (during a healthy pregnancy), which facilitates the transport of nutrients and minerals required by the foetus, via the umbilical cord. The spiral arteries are perhaps the vessels which undergo the most significant adaptation; they transform from small high resistance vessels into enlarged low resistance vessels. Approximately 200 of these spiral arteries (Burbank 2009) adapt more extensively (increasing the distal diameter, which lowers resistance), which drain directly into the intervillous space of the placenta. As with the majority of the systemic circulation, the arterial and venous sides of the utero-ovarian system are effectively symmetric. Due to a lack of information, the placenta is situated centrally in the model, which means blood will reach the placenta from both the left and right sides of the uterine circulation.

The 1D vessel network of the utero-ovarian system configuration used in the model is shown in Fig. 1d and is the most common anatomical representation of these vessels. However, a number of anatomical variations exist, such as the four different classifications given by Albulescu et al. (2014) for the origin of the uterine artery. The ovarian artery supplies blood primarily to the ovaries, while the uterine artery supplies the uterus. However, these two systems are often (not always) anastomosed via a utero-ovarian communicating artery which connects the ascending uterine artery with the ovarian artery.

Table 1 contains information on the utero-ovarian vessels used in this work, with vessel number corresponding to those in Fig. 1d. Vessel diameters of all arteries in the utero-ovarian circulation are from the literature (Dickey 1997; Espinoza et al. 2006; Burton et al. 2009; Burbank 2009; Cartwright et al. 2010; Rigano et al. 2010; Sundaram et al. 2011). There are few studies that measure the diameters of veins, and hence they are either estimated from the literature (Burbank 2009) or the diameter of the vein is assumed to be 1.25 times larger than its corresponding artery (Mynard and Smolich 2015).

In comparison with previous studies, the total length of the ascending uterine artery for participant A and B, respectively, is 10.6235 cm and 10.9268 cm, while from the literature it is 10 cm (Mo et al. 1988; Talbert 1995; Clark et al. 2018; Adamson et al. 1989). The model-estimated diameter of the uterine arteries is 2.47 mm at term, in Clark et al. (2018); Adamson et al. (1989), it is estimated to be 4 mm, and in Talbert (1995), it is estimated to be 2.8 mm (around 27 weeks gestation). The physiological ranges for uterine vessel diameters have been measured between 2 mm and 6 mm (Sundaram et al. 2011). The diameter of the arcuate artery in the model is 4 mm, while in Clark et al. (2018) it is 1–8 mm. (The effect of the vessel diameter was investigated.) The spiral arteries (for the non-pregnant case) are the vessels with the smallest diameters (0.2 mm) considered by the model.

#### Vascular beds

The vascular beds considered in this model include organs and micro-circulation at other body tissues or parts. The vascular beds are used to connect the arterial and venous networks and are located at all the terminal 1D arteries and veins shown in Fig. 1. The following organs are considered in the systemic circulation: brain, heart, liver, spleen, stomach, intestines, left and right kidneys, left and right ovaries and the uterus. The uterus is further split among three vascular beds, which are: (1) the cervix, which is the lower part of the uterus, (2) the middle part of the uterus and (3) the fundus, which is the upper part of the uterus. Additionally there are several vascular beds which represent other body regions, such as the chest, head/neck, right and left shoulders, right and left arms, pelvis, left and right upper legs, left and right lower legs. The placenta is also included in the pregnant simulation cases. In the pulmonary system, each terminal pulmonary artery is connected to a vascular bed model.

With the exception of the female reproductive organs, which have been added in this paper, the vascular bed models implemented are the same as Mynard and Smolich (2015).

#### Heart

The heart model implemented in this work is that of Mynard and Smolich (2015) and is one of the most comprehensive lumped models of the heart. The model includes all four heart chambers, four heart valves and the following three types of interaction:

- Left to right side interaction, which enables interaction between the left and right ventricles, and also between the left and right atria;
- Atria and ventricle interaction, whereby contraction of the ventricle creates a piston-like effect on the atria, which aids atrial filling;
- External pressure from the pericardium which acts on all heart chambers equally.

#### Valve

The cardiovascular model includes the four heart valves and the venous valves, which are located in the systemic veins. The valve model implemented in this work is from Mynard and Smolich (2015) and was originally proposed in Mynard et al. (2011).

The heart model contains the mitral valve between the left atria and left ventricle, the aortic valve between the left ventricle and aorta, the tricuspid valve separating the right atria and right ventricle and the pulmonary valve between the right ventricle and main pulmonary artery.

The model also includes 286 systemic venous valves for completeness, with an assumption that a valve is distributed every 4 cm in the venous system (excluding the vena cavae, which do not contain valves). Gravity is not implemented in this paper as the utilised data are for participants in the supine position, and hence, the valves have a negligible effect on the model-predicted solutions.

### Mathematical formulation

This section describes the various modelling components in the blood flow model, which includes the 1D vessel domain, the vascular bed model, the heart model and the valve model.

#### One-dimensional blood flow modelling

The nonlinear system of equations which govern 1D blood flow in a compliant vessel are the continuity equation

1 $$\begin{aligned} \frac{\partial A}{\partial P} \frac{\partial P}{\partial t} + \frac{\partial Q}{\partial x} = 0, \end{aligned}$$

where $\frac{\partial A}{\partial P}$ is the compliance, *A* is the area of the cross section, *P* is the pressure, *Q* is the volumetric flow rate, *t* is time and *x* is the spatial coordinate. The conservation of momentum

2 $$\begin{aligned} \frac{\rho }{A} \frac{\partial Q}{\partial t} + \frac{\rho }{A} \frac{\partial \left( \frac{Q^2}{A}\right) }{\partial t} + \frac{\partial P}{\partial x} + \frac{\xi \mu \pi Q}{A^2}= 0, \end{aligned}$$

where $\rho$ is the density of blood, $\xi$ is the viscous resistance coefficient and $\mu$ is the blood viscosity. The system of equations is closed with the constitutive law

3 $$\begin{aligned} P-P_{0}-P_{\mathrm{ext}} = \frac{2 \rho c_0^2}{b} \left( \left( \frac{A}{A_0} \right) ^{b/2} -1 \right) + \frac{{\varGamma }}{A_0 \sqrt{A}} \frac{\partial A}{\partial P} \frac{\partial P}{\partial t}, \end{aligned}$$

where

4 $$\begin{aligned} b=\frac{2 \rho c_0^2}{P_0 - P_{\mathrm{collapse}}}, \end{aligned}$$

$P_0$ is the reference pressure, $P_{\mathrm{ext}}$ is the external pressure, $c_0$ is the wave speed at the reference pressure, $P_{\mathrm{collapse}}$ is the pressure at which vessels collapse, $A_0$ is the cross-sectional area at the reference pressure and ${\varGamma }$ is the wall viscosity coefficient. Due to an absence of gravity in the model, vessels are not expected to collapse, which allows the same constitutive law to be implemented for arteries and veins, albeit with significantly different wave speeds. The constitutive law is composed of a power law model for the elastic term and a Voigt model for the visco-elastic term and is the same as that used in Mynard and Smolich (2015). The velocity profile is assumed to be flat for the convection acceleration terms, and a profile with a small boundary layer is chosen for the viscous term (Mynard and Smolich 2015).

#### Vascular bed models

All lumped models implemented in this paper can be constructed using a combination of the following equations: a resistance element

5 $$\begin{aligned} \frac{1}{R} \left( \Delta P \right) = Q, \end{aligned}$$

where *R* is the resistance and $\Delta P$ is the pressure difference across the resistor; a compliant (capacitive) element

6 $$\begin{aligned} C \left( \frac{\partial P}{\partial t} - \frac{\partial P_{\mathrm{ext}}}{\partial t} \right) = Q + S, \end{aligned}$$

where *C* is the compliance and *S* is a source or sink term which is used to control blood volume in the system; and an inertial (inductive) element

7 $$\begin{aligned} \frac{1}{L} \left( \Delta P \right) = \frac{\partial Q}{\partial t}. \end{aligned}$$

The vascular bed models are used to represent any arterioles, capillaries and venules, and are connected to all terminal arteries and veins. The vascular bed models implemented in this paper are the same as in Mynard and Smolich (2015), which allows any number of arteries and any number of veins to connect to a single vascular bed.

The vascular beds of the utero-ovarian system are modelled with the use of five (non-pregnant) or six (pregnant) vascular beds; these are: the left and right ovaries, the fundus of the uterus, the main body of the uterus, the cervix/vagina and the placenta (pregnancy cases only).

The vascular beds (excluding coronary beds) use pressure-dependant resistances. The vascular bed resistances $R_{\mathrm{vb}}$ are calculated as

8 $$\begin{aligned} R_{\mathrm{vb}} = {\left\{ \begin{array}{ll} R_{0} \left( \frac{p_{tm0} -p_{zf} }{p_{tm} -p_{zf}}\right) ,& \quad p_{tm} > p_{zf} \\ \infty ,&\quad p_{tm} \le p_{zf}, \end{array}\right. } \end{aligned}$$

where $R_0$ is the vascular bed resistance at a reference pressure, $p_{tm0}$ and $p_{tm}$ are the initial and the current transmural pressures, $p_{zf}$ (5 mm Hg) is the pressure at which flow in the vascular bed becomes zero.

The coronary vascular beds used volume-dependant resistances and experienced external pressures from the heart ventricles. The volume in the coronary compartments are updated asmm Hg

9 $$\begin{aligned} V_{i}(t) = V_{0,i} + \int _0^t C_{\mathrm{i}} \frac{\mathrm{d}p_{tm,i}}{\mathrm{d}t'} \mathrm{d}t', \end{aligned}$$

where *V* is the volume in the compliant compartment, $V_{0,i}$ are reference volumes and $C_\mathrm{i}$ is the intramyocardial compliance. The intramyocardial resistances are then calculated as

10 $$\begin{aligned} R_{i}(t) = R_{0,i} \frac{V^2_{0,i}}{V^2_{i}} \end{aligned}$$

and

11 $$\begin{aligned} R_{m} (t) = R_{0,m} \left( 0.75 \frac{V_{0,1}^2}{V_{1}^2} + 0.25 \frac{V_{0,2}^2}{V_{2}^2} \right) , \end{aligned}$$

which is identical to that in Mynard and Smolich (2015).

The resistance and compliance values of all vascular beds for the final cardiac cycle of each simulation case are supplied in a supplementary file.

#### Heart model

The heart model implemented in this paper is the same as in Mynard and Smolich (2015). Pressure in a heart chamber can be calculated as

12 $$\begin{aligned} P = P_{\mathrm{pc}} + E_{\mathrm{nat}} \left( V-V_{0} \right) - R_{\mathrm{s}} Q + \frac{E_{\mathrm{nat}}}{E_{\mathrm{sep}}} P^*, \end{aligned}$$

where $R_\mathrm{s}$ is a source resistance, $E_{\mathrm{nat}}$ and $E_{\mathrm{sep}}$ are the native and septal elastance of a heart chamber, *V* and $V_0$ are the volume and unstressed volume of a heart chamber, $P^*$ is the pressure in the contra-lateral chamber and $P_{\mathrm{pc}}$ is the pressure from the pericardium. The source resistance is calculated as

13 $$\begin{aligned} R_{\mathrm{s}} = K_{\mathrm{s}} E_{\mathrm{nat}} \left( V-V_{0} \right) , \end{aligned}$$

where $K_\mathrm{s}$ is a constant coefficient. The model contains three types of interaction: external pressure from the pericardium

14 $$\begin{aligned} P_{\mathrm{pc}} = K_{\mathrm{pc}} {\text {exp}}\left[ \frac{V_{\mathrm{pc}} - V_{\mathrm{0,pc}}}{\Phi } \right] . \end{aligned}$$

where $K_{\mathrm{pc}}$, $V_{\mathrm{0,pc}}$ and ${\Phi }$ are pericardium constants and $V_{\mathrm{pc}}$ is the pericardial volume; left and right chamber interaction, from pressure in the contra-lateral chamber, via the term $P^*$ in Eq. 12; and a piston-like effect $\mu _{\mathrm{AV}}$ which aids atrial filling, and acts through the native elastance

15 $$\begin{aligned} E_{\mathrm{nat}} = \frac{E_{\mathrm{fw}} E_{\mathrm{sep}}}{E_{\mathrm{fw}}+E_{\mathrm{sep}}} - \mu _{\mathrm{AV}} Q_{\mathrm{V}}, \end{aligned}$$

where $Q_\mathrm{v}$ is the flow rate in the ventricle and $E_{\mathrm{fw}}$ is the free wall elastance. The septal elastance is calculated as

16 $$\begin{aligned} E_{\mathrm{sep}} = \kappa _{L} E_{fw,L} + \kappa _{R} E_{fw,R}, \end{aligned}$$

where $\kappa _{L}$ and $\kappa _{R}$ are the left and right septal elastance constants, respectively. The free wall elastance is

17 $$\begin{aligned} E_{\mathrm{fw}} = k \left[ \frac{g_1}{1+g_1} \right] \left[ \frac{1}{1+g_2}\right] + E_{\mathrm{min}}, \end{aligned}$$

which is constructed in the same way as Mynard and Smolich (2015), and

18 $$\begin{aligned} g_1 = \left( \frac{t-t_{\mathrm{onset}}}{\tau _1} \right) ^{m_1}, \quad \quad g_2 = \left( \frac{t-t_{\mathrm{onset}}}{\tau _2} \right) ^{m_2}, \end{aligned}$$

where $t_{\mathrm{onset}}$ is the onset of contraction, $\tau _1$ and $\tau _2$ are the contraction and relaxation time offsets, $m_1$ and $m_2$ are the contraction and relaxation rate constants, and

19 $$\begin{aligned} k = \left( E_{\mathrm{fw}}^{\mathrm{max}}-E_{\mathrm{fw}}^{\mathrm{min}} \right) / {\text{max}}\left[ \left( \frac{g_1}{1+g_1} \right) ,\left( \frac{1}{1+g_2} \right) \right] . \end{aligned}$$

#### Valve model

The valve model used in this paper is identical to that in Mynard et al. (2011) and Mynard and Smolich (2015), where the valve state depends on the transvalvular pressure. Valves are constructed using a Bernoulli resistance element and an inertial element

20 $$\begin{aligned} \Delta P = B Q \left| Q \right| + L \frac{\partial Q}{\partial t}, \end{aligned}$$

where *B* and *L* are the Bernoulli resistance and the inertia, respectively, given by

21 $$\begin{aligned} B = \frac{\rho }{2 A_{\mathrm{eff}}^2}, \quad L = \frac{\rho l_{\mathrm{eff}}}{A_{\mathrm{eff}}}, \end{aligned}$$

where $l_{\mathrm{eff}}$ is an effective length, and the effective orifice area is calculated as

22 $$\begin{aligned} A_{\mathrm{eff}}(t) = \left( A_{\mathrm{eff,max}}-A_{\mathrm{eff,min}} \right) \zeta (t) + A_{\mathrm{eff,min}}, \end{aligned}$$

where $A_{\mathrm{eff,max}}$ and $A_{\mathrm{eff,min}}$ are the maximum and minimum allowed orifice area and $\zeta$ is the valve state. The valve state $0 \le \zeta \le 1$ can be updated by applying the second-order backward differences for the time derivatives

23 $$\begin{aligned} \frac{\mathrm{d} \zeta }{\mathrm{d}t} = K_{\mathrm{vo}} \left( 1-\zeta \right) \Delta P, \quad \frac{\mathrm{d} \zeta }{\mathrm{d}t} = K_{\mathrm{vc}} \zeta \Delta P, \end{aligned}$$

where $K_{\mathrm{vo}}$, and $K_{\mathrm{vc}}$ are coefficients which affect the rate of valve opening and closing, respectively.

### Boundary conditions

#### 1D vessel to vessel connectivity

At junctions between two or more vessels, Lagrange multipliers are used to constrain conservation of mass and continuity of static pressure between vessels. The method is generalised and can be applied to an arbitrary number of parent and child vessels at a junction. The implementation and validation of the use of Lagrange multipliers have been performed in Carson and Van Loon (2016).

#### Connectivity to 0D elements

The connection between neighbouring 1D and 0D elements is performed by simply sharing a pressure node. An arbitrary number of 1D elements can be connected to an arbitrary number of 0D elements by using this coupling technique, which leads to conservation of the physical quantities of mass and static pressure between the 1D and 0D elements.

The connection between two or more neighbouring 0D elements is also performed by sharing a pressure node and also leads to conservation of mass and conservation of static pressure between the 0D elements. The coupling techniques described above have been rigorously tested and validated in Carson and Van Loon (2016).

### Numerical scheme

The 1D and 0D equations are written in a similar format, allowing both 1D and 0D systems to be solved using the enhanced trapezoidal rule method (ETM) (Carson and Van Loon 2016). The ETM is an implicit sub-domain collocation scheme that uses a second-order backward difference scheme for the temporal discretisation and the composite trapezoidal rule in space. The scheme has been rigorously tested for challenging 1D wave propagation problems which includes discontinuities in geometry, compliance, pressure and flow rates (Carson and Van Loon 2016; Carson 2018).

**Table 1:** Utero-ovarian artery and vein vessel information

No.	Name	Length (cm)	Area NP (${\text {cm}}^2$)	Area P (${\text {cm}}^2$)	Vascular bed
62	Ovarian artery I	3	0.0154	0.03079	
63	Ovarian artery II (×3)	2	0.0079	0.015	Ovary
64	Communicating artery	5	0.004418	0.048105	
65	Arcuate arteries I (×4)	12	0.0123	0.1257	
66	Radial/spiral arteries I [×50]	3	0.000314	0.001963	Fundus
67	Ascending uterine artery I	3	0.02405	0.048105	
68	Arcuate arteries II (×4)	12	0.0123	0.1257	
69	Radial/spiral arteries II (×50)	3	0.000314	0.001963	Uterus
70	Ascending uterine artery II	3	0.02405	0.048105	
71	Arcuate arteries III (×4)	12	0.0123	0.1257	
72	Radial/spiral arteries III (×50)	3	0.000314	0.001963	Placenta
73	Ascending uterine artery III	3	0.02405	0.048105	
74	Arcuate arteries IV (×4)	12	0.0123	0.1257	
75	Radial/spiral arteries IV (×50)	3	0.000314	0.001963	Uterus
76	Ascending uterine artery I	3	0.02405	0.048105	
77	Descending uterine artery	3	0.000314	0.001963	
78	Arcuate arteries V (×4)	12	0.0123	0.1257	
79	Radial/spiral arteries V (×50)	3	0.000314	0.001963	$\hbox {Cervix}|\hbox {Vagina}$
80	Uterine artery	8	0.02405	0.048105	
81	Vaginal artery	7	0.00307	0.04909	
270	Ovarian vein I	3	0.0767	0.7854	
271	Ovarian vein II (×3)	2	0.0123	0.0491	Ovary
272	Communicating vein	5	0.0767	0.7854	
273	Arcuate veins I (×4)	12	0.0192	0.1963	
274	Radial/spiral veins I (×50)	3	0.0124	0.0491	Fundus
275	Ascending uterine vein I	3	0.02405	0.048105	
276	Arcuate veins II (×4)	12	0.0192	0.1963	
277	Radial/spiral veins II (×50)	3	0.0124	0.0491	Uterus
278	Ascending uterine vein II	3	0.02405	0.048105	
279	Arcuate veins III [×4]	12	0.0192	0.1963	
280	Radial/spiral veins III (×50)	3	0.0124	0.0491	Placenta
281	Ascending uterine vein III	3	0.02405	0.048105	
282	Arcuate veins IV (×4)	12	0.0192	0.1963	
283	Radial/spiral veins IV (×50)	3	0.0124	0.0491	Uterus
284	Ascending uterine vein I	3	0.02405	0.048105	
285	Descending uterine vein	3	0.000314	0.001963	
286	Arcuate veins V (×4)	12	0.0192	0.1963	
287	Radial/spiral veins V (×50)	3	0.0124	0.0491	$\hbox {Cervix}|\hbox {Vagina}$
268	Uterine vein	8	0.0767	0.7854	
266	Vaginal vein	7	0.0098	0.02405	

Right and left sides are assumed to be equal in length and area. Vessel numbers correspond with those shown in Fig. 1

*NP* represents non-pregnant conditions, while *P* represents conditions in late pregnancy

### System parameters and coefficients

In this subsection, the parameters and coefficients used in the model are described.

#### Parameters from the literature

The following parameters and coefficients are from Mynard and Smolich (2015), which is a validated closed-loop 1D–0D model of the cardiovascular system. The blood viscosity $\mu = 0.035$ poise, the fluid density is $\rho =1.06$ g/$\hbox {cm}^3$, and the viscous friction coefficient is $\xi =- 22$ which corresponds with a relatively flat velocity profile with a small boundary layer. The reference pressures are shown in Table 2, and external pressures in the entire 1D network are assumed $P_{\mathrm{ext}}=0$, due to the lack of data. The initial reference wave speed for each 1D vessel segment is calculated from the diameter using

24 $$\begin{aligned} c_0^2 = \frac{2}{3 \rho } \left( k_1 \exp \left( k_2 D_0/2 \right) +k_3 \right) , \end{aligned}$$

where $D_0$ is the diameter of the vessel segment and $k_1$, $k_2$ and $k_3$ are shown in Table 3. The values of these coefficients produce normal wave speeds in large vessels with a reasonable increase in wave speed in smaller vessels. The viscous wall coefficient is calculated as

25 $$\begin{aligned} {\varGamma }= \frac{b_1}{(2 \sqrt{A/ \pi })} + b_0, \end{aligned}$$

where the coefficients in the systemic arteries are $b_1=100$ g cm/s and $b_0=400$ g/s, while for systemic arteries and pulmonary systems a constant value of ${\varGamma }=200$ g/s is chosen.

The heart chamber parameters used in the model are given in Table 4. These parameters would be expected to change between individuals; however, due to lack of data, the same cardiac parameters are used for each simulation case.

Valve parameters are given in Table 5. The parameters used for the valve models are kept the same for all simulation cases. Estimation of vascular bed parameters for the pulmonary system and the coronary circulation is performed in the same way as Mynard and Smolich (2015). However, vascular bed parameters in the systemic system are estimated using measured patient data, which are described in Sect. 2.5.2.

**Table 2:** Defined reference pressures $P_{0}$ for each system where DBP is the diastolic blood pressure from the patient measurements

Location	Pressure (mm Hg)
Systemic arteries	DBP
Systemic veins (excluding hepatic portal system)	5
Hepatic portal veins	8.5
Pulmonary arteries	11
Pulmonary veins	10

**Table 3:** Coefficients used to calculate initial wave speed $c_0$

Location	$k_1$ ($10^6\hbox { g}/\hbox {s}^2/\hbox {cm}$)	$k_2$ ($\hbox {cm}^{-1}$)	$k_3$ ($10^4\hbox { g}/\hbox {s}^{2}/\hbox {cm}$)
Systemic arteries (excluding coronaries)	3.00	$-$ 9	33.7
Systemic veins (excluding coronaries)	0.60	$-$ 5	2.8
Coronary arteries	20.00	$-$ 22.5	86.5
Coronary veins	4.44	$-$ 22.5	19.2
Pulmonary arteries	1.30	$-$ 7	12.2
Pulmonary veins	0.29	$-$ 5	2.1

**Table 4:** Cardiac parameter values where LV is the left ventricle, LA—left atria, RV—right ventricle, RA—right atria and *T*—the cardiac period

	LV	LA	RV	RA
$E_{\mathrm{min}}$ $\left( \hbox {mm Hg ml} \right)$	0.07	0.09	0.035	0.045
$E_{\mathrm{max}}$ $\left( \hbox {mm Hg ml} \right)$	2.8	0.13	0.45	0.09
$\tau _1$ $\left( \hbox {s}\right)$	0.26875 T	0.0525 T	0.26875 T	0.0525 T
$\tau _2$ $\left( \hbox {s}\right)$	0.5025 T	0.1725 T	0.5025 T	0.1725 T
$m_1$	1.32	1.99	1.32	1.99
$m_2$	21.9	11.2	21.9	11.2
$V_{0}$ $\left( \hbox {ml} \right)$	10	3	40	7
$V_{t=0}$ $\left( \hbox {ml} \right)$	136	71	172	67
$K_{\mathrm{s}}$ $10^{-3} \left( \hbox {ml} \right)$	0.5	0.25	1	0.5
$\kappa$	6	2	6	2
$\mu _{\mathrm{AV}}$ $\left( \hbox {g cm}^{-7}\, \hbox {s}^{-1} \right)$	0	0.033	0	0.05
$t_{\mathrm{onset}}$ $\left( \hbox {s}\right)$	0	0.8125 T	0	0.8125 T

**Table 5:** Valve parameter values where AV is the aortic valve, MV—mitral valve, PV—pulmonary valve, TV—tricuspid valve, VV—venous valve and $A_{\mathrm{con}}$ —the cross-sectional area of the connecting vessel

	AV	MV	PV	TV	VV
$A_{\mathrm{eff,max}}$, $\left( \hbox {cm}^{2} \right)$	6.9	5.1	5.7	6.0	$A_{\mathrm{con}}$
$A_{\mathrm{eff,min}}$, $\left( \hbox {cm}^{2} \right)$	0	0	0	0	0
$l_{\mathrm{eff}}$, $\left( \hbox {cm} \right)$	1.5	2	1.5	2	1
$K_{\mathrm{vo}}$, $\left( \hbox {cm}^{2}\,\hbox {s}^{2}\,\hbox {g}^{1} \right)$	0.02	0.02	0.02	0.03	0.03
$K_{\mathrm{vc}}$, $\left( \hbox {cm}^{2}\,\hbox {s}^{2}\,\hbox {g}^{1} \right)$	0.02	0.04	0.02	0.04	0.03

#### Parameters from measured participant data

The measured participant data utilised in this paper are primarily from Carpenter et al. (2015a, b, c, 2016a, b) and D’Silva et al. (2013). Measurements were performed using the Task $\hbox {Force}^{\copyright }$ monitor by CNSystems (Graz, Austria). The monitor includes:

- An impedance cardiograph (ICG).
- An electrocardiogram (ECG).
- Continuous ‘beat-to-beat’ blood pressure measurement using finger plethysmography.
- Oscillometric blood pressure measurement using an automated brachial cuff system.

The device allows the following beat-to-beat variables to be measured or estimated:

- Systolic, diastolic, mean and pulse blood pressures in the systemic arterial system (brachial artery).
- Heart rate and stroke volume (and hence cardiac output).
- Total peripheral resistance (TPR) (systemic vascular resistance).
- Left ventricular ejection time (LVET).
- Baroreceptor reflex sensitivity.

The measured data utilised in the current model include: systolic and diastolic pressures, heart rate and cardiac output. The model utilises an initial and adaptive parameter estimation technique described in Carson (2018) to adapt several model parameters in the systemic circulation, enabling the model to converge towards the cardiac output, systolic and diastolic pressures from the measured participant data. The measured participant heart rate is used as a model input; systemic vascular resistances in the model are iteratively adapted to converge the model-predicted mean pressure to the participant measured mean pressure in the brachial artery; the model systemic artery compliances are iteratively adapted to converge the model-predicted pulse pressure to the measured pulse pressure; and blood volume is iteratively added or removed from the system to converge to the models stroke volume to the measured stroke volume and hence converge to the measured cardiac output.

The LVET from the measured data is not used in the simulation, as through various tests on the model, the LVET has a negligible effect on the solution. The participant height is used as a scaling factor for all vessel lengths *L* by using the relation $L=\gamma L_{\mathrm{base}}$, where $L_{\mathrm{base}}$ are the vessel lengths expected in a participant with a height of 6 ft 1 inch (from Mynard and Smolich (2015)) and $\gamma$ is the scaling factor given by

26 $$\begin{aligned} \gamma = \frac{{\text {Participant height}}}{{\text {Base height}}}. \end{aligned}$$

Participant age and height are given in Table 6.

In such a complex system, one of the main difficulties is in choosing initial conditions and parameter estimation. In the model, the initial flow rates are considered to be zero in the entire system, while initial pressures in each system are chosen to be equal to the reference pressures $P_0$. As an initial assumption, the compliance of the systemic veins is chosen to be 30 times larger than that of the systemic arteries; additionally, no data on the pulmonary system were available; hence, pulmonary vascular bed resistances are scaled to achieve mean pressures which are considered to be in the ‘healthy’ range.

The initial resistances in the systemic arterial system are first estimated using the mean arterial pressure (MAP) and cardiac output (CO) from the measured participant data. To estimate the systemic vascular resistance (total peripheral resistance) for the model, the common relation (Hill et al. 2013) is used

27 $${{\text{TPR}}} = \frac{{{\hbox{MAP}}}}{{{\hbox{CO}}}}.$$

The total peripheral resistance is distributed using the estimated percentage ($\alpha$) of cardiac output to each region (van de Vosse and Stergiopulos 2011)

28 $$\begin{aligned} R_{\mathrm{region}} = \alpha {\text {TPR}}, \end{aligned}$$

where $R_{\mathrm{region}}$ is the resistance of a region. Finally, for regions containing multiple vascular beds, the expected flow distribution in the region is estimated using Murray’s law of bifurcations (Murray 1926), which uses the principle of minimum work to give a relationship between parent and child vessel radii.

The initial model compliance ($C_T$) in the systemic arterial system is estimated by assuming the relationship $\tau ={\text {TPR}} \times C_T$, where $\tau =1.79$. The compliances in the vascular beds are then found by subtracting the 1D compliances $C_{\mathrm{1D}}$ from the total compliance (van de Vosse and Stergiopulos 2011)

29 $$\begin{aligned} C_{\mathrm{0D}}=C_T - C_{\mathrm{1D}}. \end{aligned}$$

The compliances in the vascular beds are then distributed using the inverse relationship to that of resistance.

Blood volume is incrementally added (or removed) from the system via the compliance element representing the venules (in both pulmonary and systemic systems). This can be done as the compliance element is originally derived from conservation of mass, and hence, a small volume *S* can be added to the system using Eq. (6).

#### Assumptions of the model

However, the majority of estimates for parameters, coefficients and vessel network information are based on those in the literature. There are several assumptions which are required due to lack of data. The lengths of vessels are assumed to change based on the participant height; however, vessel diameters are assumed to not change based on the participant information. We have assumed that the distribution of systemic vascular resistance to each vascular bed is based on a combination of expected flow distribution to each region and distal vessel diameters (Carson 2018). When iterative adaptation of 1D vessel compliances is performed, the wave speeds in all vessels are scaled by the same amount, which means the distribution of the compliance in the 1D vessel network remains the same. During the iterative process of adding blood volume to the venules, the added blood volume is equally distributed to each vascular bed throughout the entire network.

The simulation terminates when two conditions are satisfied: (1) the model gives pressures and cardiac output within $\pm 0.5\%$ of the measurements (convergence to measured data) and (2) the solution of the final cardiac cycle has changed by less than 0.5% of the previous cardiac cycle (periodic convergence of the model).

### Methods used in analysis

In this subsection, a description of the various calculated parameters used to compare and validate the model after the simulation is presented. The pulse wave velocity (PWV) is a common measure to estimate arterial stiffness (Obeid et al. 2017). There are a few variations in pulse wave velocity determination, such as the carotid–femoral, heart–ankle, brachial–ankle and finger–toe methods. PWV effectively measures the average velocity of the pulse wave between two positions. However, in the model the PWV is much easier to calculate, as a mathematical expression can describe the wave speed at any point in the system via the characteristic system (Carson 2018); normally, this would equate to the forward travelling wave. To derive the model estimation of PWV, consider the characteristic system which can be written as

30 $$\begin{aligned} \frac{\partial w_{\pm }}{\partial t} + \lambda _{\pm } \frac{\partial w_{\pm }}{\partial x} = 0, \end{aligned}$$

where *t* is the time, *x* is the spatial coordinate in the axial direction, $w_{\pm }$ represents either the forward (+) or backward (−) characteristic variable and $\lambda _{\pm }$ represents the propagation speed of the mathematical system from Eqs. (1), (2) and (3) in either the forward (+) or backward (−) direction. The propagation speed in the forward and backward direction can be written in terms of the wave speed *c* and the velocity of blood *u* as

31 $$\begin{aligned} \lambda _{\pm } = u \pm c. \end{aligned}$$

Equation (31) clearly shows that the propagation speed of the system will depend on both time and space, as both *u* and *c* have spatial and temporal dependence. The true pulse wave velocity would be $PWV=\lambda _+$. However, the PWV can also be estimated using the initial wave speed by assuming $u=0$ and $c=c_0$, which equates to the wave speed at the diastolic pressure under the assumption that the blood velocity is negligible during diastole. Thus, for simplicity, the estimate of PWV used in this paper is ${\text {PWV}}=c_0$.

In clinical practice, it is impossible to measure the local aortic pulse wave velocity non-invasively. One of the methods which have been used to estimate aortic PWV is the brachial–ankle ${\text {PWV}}_{b-a}$ (Oyama-Kato et al. 2006). This estimation can also be performed on the model output by

32 $$\begin{aligned} {\text {PWV}}_{b-a} = \frac{L_{b}-L_{a}}{\Delta t_{b-a}}, \end{aligned}$$

where $L_{b}$ and $L_{a}$ are the distances from the aortic valve to brachial artery and ankle (anterior tibial artery), respectively, as estimated by Oyama-Kato et al. (2006), while $\Delta t_{b-a}$ is the time difference between the initial rise in pressure (beginning of systole), in the brachial artery and anterior tibial artery.

There are several ways to estimate mean arterial pressure, which in turn is used to estimated total peripheral resistance, and hence, we present the three estimations utilised: (1) an implicit estimation by the measurement device, (2) the mean pressure estimated by the model and (3) a common estimation based on systolic and diastolic pressures. The monitoring device uses the following equation to calculate the total peripheral resistance (in mm Hg min/L)

33 $${{\text{PTPR}}} = \frac{{{{\text{MABP}}}}}{{{{\text{CO}}}}} \cdot 80,$$

where 80 is a unit conversion factor, where the mean arterial blood pressure MABP is implicitly calculated by the measurement device using

34 $$\begin{aligned} {\text {MABP}}=\frac{1}{{\text {RR}}} \int _{{\text {QRS}}_{t_{\mathrm{start}}}}^{\text {QRS}_{t_{\mathrm{end}}}} p(t) \phantom {.} \mathrm{d}t, \end{aligned}$$

where the *RR* interval is ${\text {RR}}={\text {QRS}}_{t_{\mathrm{end}}} - {\text {QRS}}_{t_{\mathrm{start}}}$ and CO is the cardiac output.

In the model, a different method to estimate *MABP* (as the model does not have or use an ECG signal) is given by

35 $$\begin{aligned} {\text {MABP}} = \frac{1}{N} \sum _{i=1}^{N} P_{i}, \quad {\text {for}} \quad i=1 \ldots N, \end{aligned}$$

where *N* is the total number of time points and *P* is the pressure at the centre point of the vessel (left brachial artery).

The third method, which is a common way of estimating MABP, is by estimating the mean arterial pressure using diastolic and systolic pressures (used in clinical practice, when only oscillometric blood pressure measurements are performed), which results in the equation (Hill et al. 2013)

36 $$\begin{aligned} {\text {TPR}}=\frac{\frac{2}{3}({\text {DBP}})+\frac{1}{3}({\text {SBP}})}{{\text {CO}}} \cdot 80, \end{aligned}$$

which can be estimated for both the measured data and the model-predicted solution.

The pulsatility index (PI) determines the velocity variability in a vessel and is calculated using

37 $$\begin{aligned} {\text {PI}} = \frac{u_{\mathrm{max}}-u_{\mathrm{min}}}{u_{\mathrm{mean}}}, \end{aligned}$$

where $$u_{\mathrm{max}}$$$$u_{\mathrm{min}}$$ and $$u_{\mathrm{mean}}$$ are the maximum, minimum and mean velocities, respectively. In the model, the velocity (including maximum velocity) is defined as $$u=\frac{Q}{A}$$.

**Table 6:** Participant information

	Participant A	Participant B
Age	36	28
Height	164.6 cm	168 cm

### Numerical experiments of the closed-loop model

The flow rates to the uterine region of the current model have been validated in Carson (2018). Furthermore, a sensitivity analysis performed in Carson (2018) indicated that the adaptive parameter estimation technique is insensitive to the initial conditions of the model.

## Results

### Model comparisons with non-invasive *in vivo* measurements

**Fig. 2:**
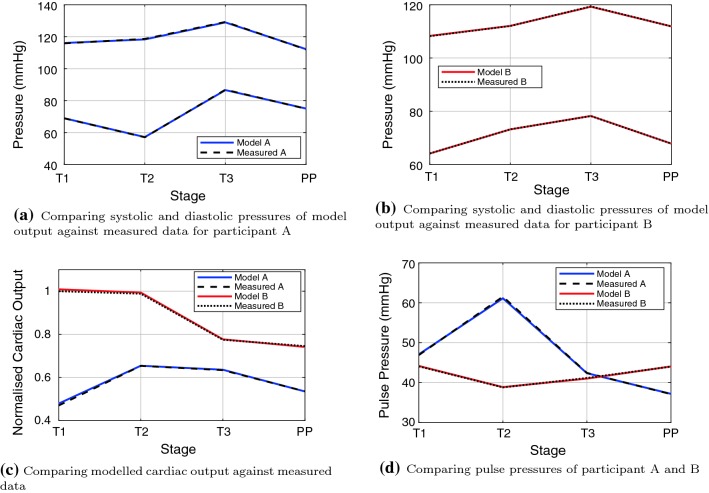
Comparison of model outputs with participant measurements over the three stages of pregnancy, trimesters 1–3 (*T*1, *T*2, *T*3) and for post-pregnancy (PP). Model A and Measured A are the model-predicted and measured data for participant A, respectively. Model B and Measured B are the model-predicted and measured data for participant B, respectively

Figure 2 shows a comparison of the model-predicted solutions with measured haemodynamic quantities. With the proposed optimisation strategy, the model is perfectly capable of reproducing the measurements for the two participant case studies. Moreover, Table 7 shows the sensitivity of the final solutions with regard to initial pressures. The sensitivity test shows a comparison of two simulations of participant B in the third trimester (as this requires a larger adaptation than the other cases and hence will be a more difficult test for the iterative scheme). Given that the initial pressures for the second simulations are increased by 10, the system is insensitive to initial pressures.

The two participants have significant differences in cardiac outputs as shown in Fig. 2c with participant B having approximately double the cardiac output of participant A in trimester 1. Moreover, the cardiac output for the two individuals follows different trends over time; for B, the largest value occurs during the first trimester and shows a large reduction by the third trimester; A follows a more typical behaviour in time, with an increase in cardiac output from trimester 1 to 2 and then a reduction during trimester 3.

**Fig. 3:**
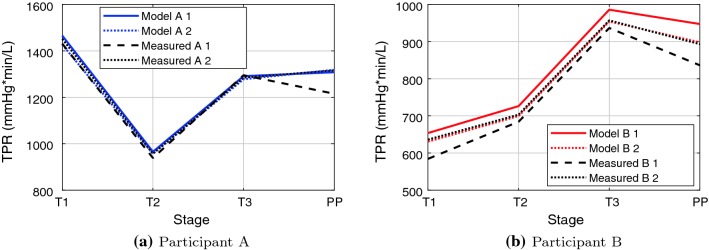
Comparison of model-derived total peripheral resistance (TPR), with measured participant data. Participant A is shown in 3a, and participant B is shown in 3b. Model 1 uses Eq. (35), Measured 1 uses equation (34), and Model 2 and Measured 2 use equation (36)

The systolic and diastolic blood pressures for participant A are shown in Fig. 2a. The pulse pressure, which is shown in Fig. 2d, increases from 47 mm Hg in trimester 1 to a large pulse pressure of 61 mm Hg in trimester 2; this is an indication of inadequate levels of adaptation, where arterial compliance has not increased to an acceptable level to accommodate the extra blood volume in the system. However, the pulse pressure reduces by the third trimester to a normal/expected pulse pressure of 43 mm Hg, indicating that although physiological adaptation occurred more slowly than ideal, the correct level of adaptation did eventually take place. Post-natally the pulse pressure reduced further to 37 mm Hg.

Systolic and diastolic blood pressures for participant B are shown in Fig. 2b, and the pulse pressures are shown in Fig. 2d. The mean arterial pressure for participant B increases by approximately 10% from trimester 1 through to trimester 3; however, these pressures are still in the physiologically healthy range. Moreover, the pulse pressures for participant B remain relatively stable over the course of pregnancy, following the expected behaviour for a healthy pregnancy, with a decrease from 44 mm Hg in trimester 1 to 39 mm Hg in trimester 2, followed by a small increase to 42 mm Hg in trimester 3. The pulse pressure increases to 44 mm Hg post-natal.

The total peripheral resistances (TPR) are shown in Fig. 3. There are two alternative representations for the TPR for both the measured and model-estimated resistances. Representation 1 for the measured data uses Eqs. (33) and (34); variation 1 of the model uses Eqs. (33) and (35), while variation 2 for both measured data and the model use equation (36). The TPR is larger for participant A than for participant B for all three trimesters and post-pregnancy. The behaviour is also significantly different between participants; it would be expected that the resistance would decrease between trimesters one and two, and be relatively stable from trimesters two to three, followed by an increase in TPR post-pregnancy (Burbank 2009). Participant A experiences a large decrease in resistance between the first and second trimesters, with an increase in TPR between trimesters two to three, while participant B experiences an increase between trimester one and two, and a further increase to trimester three. Measured and model-estimated TPR was in excellent agreement when using representation 2 (as would be expected as the automated iteration converges for CO, SBP and DBP); the model-estimated TPR using Eq. (33) follows the same trend as that using representation 2, with the main difference between the methods being the estimation of mean arterial pressure. However, representation 1 of the measured data (Eq. 33) follows a slightly different trend for participant 1, showing a decrease in TPR post-pregnancy, while all other estimations show an increase in TPR. The magnitudes of the TPR of all methods are quite similar.

The results up to this point have essentially compared the model (and its automated parameter adaptation), with the measured data. Hence, it has mainly showed the effectiveness of the model’s parameter estimation and iteration. However, the remainder of the results presented use the novel, model-predicted solutions to provide additional insight.

**Table 7:** Model sensitivity to initial pressures

Parameter	MAPE
Aortic pressure	0.0701
Aortic flow	0.0677
Left brachial artery pressure	0.0675
Left brachial artery flow	0.0202
Left uterine artery pressure	0.0015
Left uterine artery flow	0.1557
Left communicating artery pressure	0.0592
Left communicating artery flow	0.1879

Case comparison uses two simulations for participant B during the third trimester, where one simulation has increased initial 1D pressures by 10%. Mean absolute percentage errors (MAPE) are shown for the centre of the respective vessels

### Pulse wave velocity and pulsatility index

**Fig. 4:**
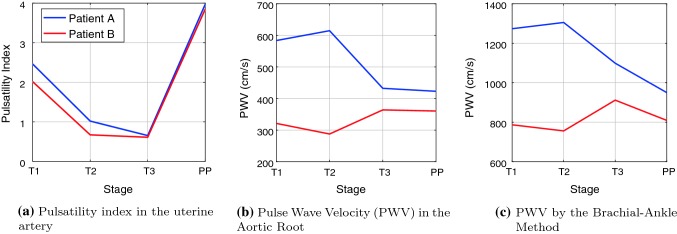
Model-derived parameters for both participants over the three trimesters and post-partum

Figure 4c shows the pulse wave velocity (PWV) in the ascending aorta, estimated using Eq. (31). The model-predicted PWV for participant B follows the expected general trends as described by Oyama-Kato et al. (2006).

Participant A follows a significantly different trend than participant B, during the first and second trimesters; the PWV increases from the first to second trimester for participant A, while PWV significantly decreases from trimester two to three. The PWV is much higher in the first and second trimesters for participant A, compared to participant B, although values become closer in the third trimester and in post-pregnancy. The result of the brachial–ankle technique, which estimates PWV is shown in Fig. 4d, and uses Eq. (32). Both estimations of PWV (aortic and brachial–ankle) from the model follow a similar trend, although their magnitudes are significantly different. The magnitudes of the brachial–ankle PWV are closer to the estimates given in Oyama-Kato et al. (2006) than the magnitudes calculated via the aortic PWV.

An additional measure often linked to arterial stiffness is the pulsatility index (PI) (Gómez et al. 2008; Guedes-Martins et al. 2015), which is shown in Fig. 4b. The pulsatility index (given by equation (37)) of the model is in agreement with expected values (Gómez et al. 2008) at the end of the first trimester PI$\in {[1.2,2.7]}$, PI$\in {[0.7,1.5]}$ at the end of the second trimester and PI$\in {[0.5,1.0]}$ at the end of the third trimester. Moreover, the pulsatility index for the post-pregnancy data is in agreement for those measured in Guedes-Martins et al. (2015), which measured the pulsatility index over the entire menstrual cycle, with normal values in the range PI$\in {[1.0,5.5]}$.

### Uterine artery waveforms

**Fig. 5:**
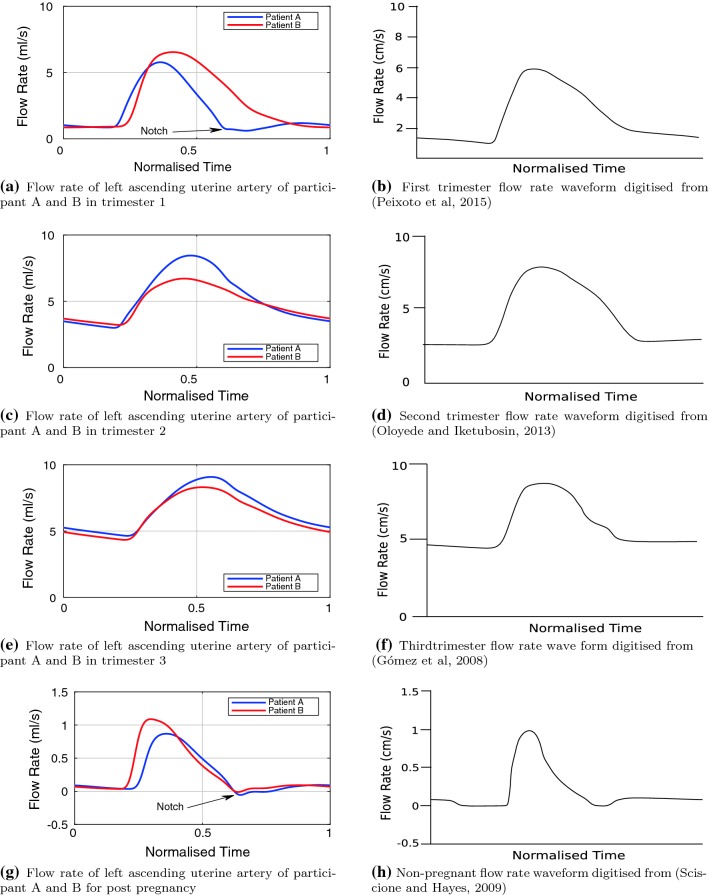
Comparison of participant A and B left ascending uterine artery flow rate waveforms and scaled published Doppler waveforms. Normalised in time and digitised Doppler waveforms are scaled by physiologically realistic uterine artery area to aid comparison

As mentioned in Sect. 2.1.2, the placenta is centrally located in the model and the 1D utero-ovarian network is symmetric; this causes the solutions in the left and right arteries in the utero-ovarian circulation to be identical. The uterine artery waveforms obtained from the model for participants A and B are shown in Fig. 5. The uterine artery waveform shape is in agreement with those given in the literature for all pregnant and non-pregnant cases, where Doppler waveforms in the non-pregnant cases show notching at the end of systole, and close to zero flow during diastole, as shown in Fig. 5h, while during pregnancy any notching at end systole is dampened out of the waveform due to the increase in compliance. There are also significant diastolic flow rates, particularly during the second and third trimesters. Typically these waveforms are measured via pulsed Doppler ultrasound (Khong et al. 2015; Sciscione and Hayes 2009).

The model output for the non-pregnant waveforms is shown in Fig. 5g, with a close to zero diastolic flow rate, and notching in the waveforms around $t=0.6$, where the flow briefly becomes negative. The waveforms from the model show similar behaviour as the digitised waveform from a Doppler study (Sciscione and Hayes 2009).

The computed first trimester waveforms are shown in Fig. 5a. In the flow waveform for participant A, a small amount of notching is still present, while for participant B, no notching is seen. The notching in participant A’s waveform may indicate an elevated level of arterial stiffness. However, this does not imply an abnormal pregnancy, as notching in the uterine artery waveform was observed for 46.3% of woman in weeks 11–14 (Gómez et al. 2008). The shape of the first trimester waveform of participant B shows excellent agreement with the waveform from a Doppler study (Peixoto et al. 2015), shown in Fig. 5b. Notching is no longer present in the second trimester as shown in Fig. 5c. Again the shape of the waveforms is in good agreement with the digitised waveform from a Doppler study (Oloyede and Iketubosin 2013), shown in Fig. 5d. For the third trimester, the model-predicted flow waveforms for participant A and B are quite similar, with a greater mean flow rate for participant A. The shape of both waveforms is in good agreement with the digitised waveform from a Doppler study (Gómez et al. 2008), shown in Fig. 5f.

### Uterine vein waveforms

**Fig. 6:**
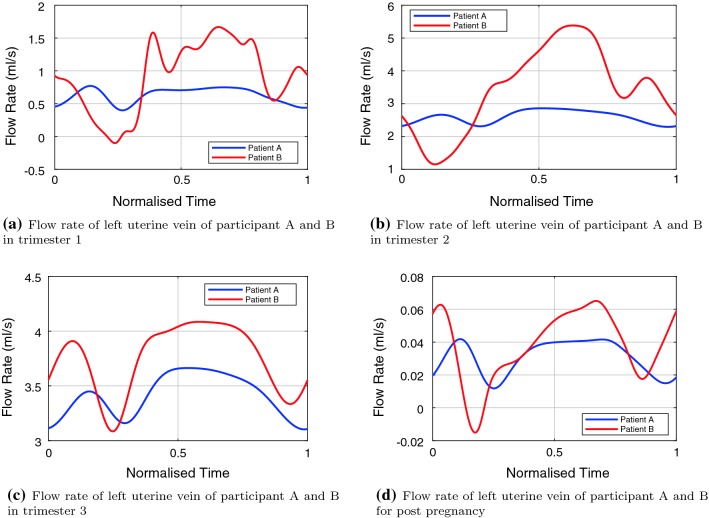
Comparison of participant A and B left uterine vein flow rate waveforms. Normalised in time for comparison

The uterine vein waveforms for participants A and B are shown in Fig. 6. The waveforms of participant B show more pulsatility than those of participant A for all trimesters and for the post-pregnant case. The waveform shapes are in agreement with the three common types of uterine vein waveforms measured in Thuring et al. (2010).

The model output for participant A shows a low pulsatile, continuous flow in all trimesters, while participant B shows significant pulsatility, with flow approaching zero and becoming negative in the first trimester waveform in Fig. 6a and the post-pregnant waveform in Fig. 6d.

### Flow distribution to the uterus

The flow distribution to the uterus changes significantly during pregnancy and is an important factor in estimating the resistance of the uterine system. The model-predicted percentage of cardiac output reaching the main body of the uterus and the placenta is given in Table 8. Table 9 shows published values for the blood flow to the uterus. Hale *et al.* (Hale et al. 2009) estimated the percentage of cardiac output reaching the uterus in non-pregnant women to be $0.45 \pm 0.19 \%$, agreeing well with the model output. Total flow in the uterine arteries (sum of left and right ascending) of participant A is 0.54%, with a further 0.01% from the utero-ovarian arteries, while for participant B the left and right combined blood flow through the ascending uterine arteries is 0.48%, with an additional 0.006% from the utero-ovarian communicating arteries.

Although significant variation exists between individuals, Thaler et al. (1990) estimated that the percentage flow (bilateral) of cardiac output in the ascending uterine arteries is approximately 3.5% in early pregnancy, raising to approximately 12% by term. The model output for participant A gave the percentage of cardiac output to the uterus via the ascending uterine arteries in the first trimester as 5.44%, increasing to 12.58% by the third trimester, with a further contribution of 4.52% from the ovarian communicating arteries, increasing to 9.46% by the third trimester. In terms of actual flow rate via the uterine artery, the model is in the correct physiological range with 0.25 l/min in the first trimester and 0.785 l/min in the third trimester (Konje et al. 2001; Osol and Mandala 2009).

For participant B, the model gives a percentage cardiac output to the uterus from the ascending uterine arteries of 3.42%, increasing to 9.68% for the third trimester, with a further contribution of 3.08% (in the first trimester) increasing to 7.46%, respectively, from the utero-ovarian communicating artery. The volumetric flow rate to the uterus from the ascending uterine arteries is 0.338 l/min in the first trimester and 0.738 l/min in the third trimester.

Previous attempts to measure the contribution of blood volumetric flow rates to the uterus via the utero-ovarian communicating arteries have been unsuccessful (Pates et al. 2010), mainly due to the complexity of blood flow in the pelvic region (Browne et al. 2015). Hence, the model described here can give an estimation of previously unknown/unmeasured volumetric flows to the uterus.

**Table 8:** Percentage of cardiac output to each vessel

	Participant A				Participant B			
	*T*1	*T*2	*T*3	Post	*T*1	*T*2	*T*3	Post
Ascending uterine artery	2.72	4.91	6.29	0.27	1.71	2.96	4.84	0.24
Communicating artery	2.26	3.75	4.73	0.005	1.54	2.30	3.73	0.003

Flows in the left and right sides are the same in the model

**Table 9:** Flow to uterus from the uterine arteries from the literature (where available)

	Status	Flow rate	Percentage of CO	References
Bilateral ascending uterine artery	Early pregnancy	–	3.5	Thaler et al. (1990)
Bilateral ascending uterine artery	At term	–	12	Thaler et al. (1990)
Bilateral uterine artery	Non-pregnant	20–50 ml/min	–	Osol and Mandala (2009)
Bilateral uterine artery	Singleton pregnancies	450–800 ml/min	–	Osol and Mandala (2009)
Bilateral uterine artery	Week 36	700 ml/min (estimated 921 at term)	–	Osol and Mandala (2009)
Bilateral uterine artery	Week 20	531 ml/min	–	Konje et al. (2001)
Bilateral uterine artery	Week 38	970 ml/min	–	Konje et al. (2001)

## Discussion

An automated modelling framework was successfully developed that can integrate the multi-variate pregnancy measurements as collected by the group of M. Lewis (Carpenter et al. 2015c, a, b, 2016a, b; D’Silva et al. 2013). The cardiovascular network models are able to consistently reproduce measured variables such as pulse pressure, total peripheral resistance and cardiac output. After tuning the models to the measurements (using an automated process), the models provide predictions of variables that have not been measured such as uterine arterial/venous flow and pulse wave velocity. These variables compare favourably with the physiological ranges observed throughout pregnancy.

### Participant A and participant B comparison

Participant A was eight years older than participant B so the observed differences in behaviour in early pregnancy could be expected, as age is known to be an important factor with regard to cardiovascular adaptation, maternal health during pregnancy and pregnancy outcomes (Salmi et al. 2010).

The cardiac output for participant A follows the expected trend, with an increase in CO from trimester one to trimester two. With this increase in CO, the pulse pressure increases significantly from 47 mm Hg in trimester one to 61 mm Hg in trimester two. Elevated pulse pressure has been linked with an increased risk of gestational hypertension and pre-eclampsia (Thadhani et al. 2001). However, this link is generally associated with elevated mean arterial blood pressures. Participant A had relatively healthy mean pressures, with normal systolic pressure and relatively low diastolic pressure. This indicated that participant A had normal total peripheral resistance, while having slightly elevated arterial stiffness. It could be anticipated that physiological adaptation, especially that relate to compliance, would occur more slowly for an older individual, as studies have indicated increased arterial stiffness in older pregnant women (Salmi et al. 2010) and also in non-pregnant individuals (Lee and Oh 2010).

Participant B shows an unusual trend in CO, with a significant reduction between trimesters two and three. Furthermore, the pulse pressure remained relatively stable around 40 mm Hg, while the mean arterial pressure increased by approximately 5 mm Hg. This indicated that there was an increase in both total peripheral resistance and arterial stiffness. The cause of this unusual behaviour is unknown, but we might speculate that it is related to dehydration, which is known to cause reduced cardiac output, while increasing vascular resistance (Gonzalez-Alonso et al. 1995). Moreover, during pregnancy every aspect of renal function is affected (Cheung and Lafayette 2013), increasing the risk of dehydration, although dehydration would normally cause a drop in pulse pressure. An alternative and more likely cause (due to the extreme change in pulse pressure) of this abnormal behaviour is that the compliance of the systemic veins could have increased significantly between trimesters two and three. This would provide more storage capacity for blood, reducing venous return and hence cardiac output. However, no measurements on the venous system were taken, so it is conjecture as to the cause of this unusual behaviour of the cardiac output.

The model has shown to be effective in capturing participant-specific haemodynamic quantities. The model results closely follow the measured data over a range of physiological conditions. Moreover, the model is insensitive to the defined initial pressures (initial blood volume).

### Comparison with previous studies

One of the difficult challenges in modelling such a complex system is the extreme variation in haemodynamic quantities and even measured vessel sizes reported in the literature. For example, the diameter of the uterine artery has been reported to be between 1.4 and 5 mm for a non-pregnant woman and 4.8 and 7 mm at term. Bilateral blood flow in the uterine arteries has been reported between 18 and 180 ml/min for non-pregnant individuals and 492 and 970 ml/min in singleton pregnancies at term, with similar variations seen for other vessel diameters and flow rates (Burbank 2009). Our model estimates of mean flow rates through the uterine arteries are close to reported mean values, with values for participants A and B of 785 ml/min and 738 ml/min, respectively. The model also provides estimates of flow rates to the uterus from the utero-ovarian communicating arteries for the first time. The model assumed the diameters of the utero-ovarian communicating arteries were the same as those of the ascending uterine arteries at term (Burbank 2009). Using this assumption, the total flow through the utero-ovarian arteries for participants A and B at term was 600 ml/min and 570 ml/min for participant A and B, respectively. Given that the utero-ovarian communicating arteries are capable of supplying the uterus with its full blood supply needs, when the vessel diameter matches that of the ascending uterine artery (Burbank 2009), it is reasonable to assume their blood flow rate will also be close to that of the ascending uterine arteries, which the model predicts. However, this will depend on the assumption of the adaptation of vessel diameter.

A Doppler ultrasound assessment of uterine artery blood flow is routinely performed on participants at high risk of developing pathologies such as pre-eclampsia. The technique is used to detect abnormalities in flow pattern, such as the volumetric flow rate (which is linked to the resistance of the utero-placental system) or the presence of notching in the flow waveform (an indication of elevated arterial stiffness, caused by reduced compliance of the uterine vessels or the placenta). Another use of the Doppler assessment is to determine the pulsatility index, as a high PI in the uterine artery is thought to indicate increased impedance to flow caused by poor placentation (poor connection of the placenta to the uterus, which is linked to both placental insufficiency and pre-eclampsia). The model gave values of PI within the healthy range reported in the literature and showed the expected trend for a healthy pregnancy for both participants tested (Gómez et al. 2008; Tayyar et al. 2015). Moreover, the flow waveforms showed good agreement with those from previous Doppler studies for all three trimesters and for the post-pregnancy cases.

### Limitations

In this paper, only measured data from systemic arteries were available and so the model assumes healthy conditions in the systemic veins and in the pulmonary system. Moreover, there are a large number of possible variations in cardiovascular network anatomy between individuals. In this work, the most common utero-ovarian network configuration was chosen. Furthermore, the placenta was assumed to be connected to the same location in the uterus for all simulations. The distribution of resistances was based on estimations for the percentage of cardiac output to each body region, which for the model is assumed to be the same for both individuals due to lack of data, but in real physiological conditions will vary between individuals.

The arterial compliance changes differentially in the uterine vessels in comparison with other vessels as a result of several factors, including placentation, foetal growth and changes in hormonal levels (particularly oestrogen and progesterone) (Osol and Mandala 2009; Mandala and Osol 2011). However, this information is not known for participants in this study. The amount of compliance distributed to the utero-ovarian circulation increased as the model progresses towards term in pregnancy. The distribution of compliance in the 1D network was assumed to be the same for each participant, although the initial distribution of compliance changed whether the model was simulating the first trimester, second trimester, third trimester or post-partum cases. However, once the initial distribution of compliance is determined, any percentage change in systemic arterial compliance, as calculated by the automated parameter adaptation, is distributed equally within the 1D system.

Parameters of the heart model (such as elastance curves) were assumed to be the same for all simulations, although the heart does remodel during pregnancy. However, in the model, heart function does implicitly change for each simulation as preload is affected by the volume of blood, after-load is affected by pressures in the arteries, and contractility is affected through changes in heart rate. (In the model the rate of change in contraction force varies with heart rate.)

In addition, the estimation of flow rate through the utero-ovarian arteries cannot be directly validated, as attempts to use modern non-invasive techniques to measure these arteries have currently been unsuccessful (Pates et al. 2010; Browne et al. 2015). However, it has been mentioned that if the utero-ovarian communicating arteries adapt to be the same diameter as the ascending uterine arteries, then they can supply the uterus with its full blood supple needs (Burbank 2009).

No flow or pressure waveforms were taken from the participants; hence, the models waveforms have only been compared to digitised waveforms from published Doppler studies. As the shape of our models' waveforms is in good agreement with those found in the literature, the volumetric flow rates through the uterine arteries are in physiologically realistic ranges, and the pulsatility indices are in physiological ranges for all trimesters and post-pregnant cases, this indicates that the model-predicted waveforms are physiologically representative.

As a result of these assumptions, one has to be cautious when interpreting the modelling results. Nevertheless, the results presented in this work show that the numbers are consistent with medical literature, which gives confidence when using these models to study flow physiology on a patient-by-patient basis.

## Conclusion

In this paper, a closed-loop 1D–0D model of the maternal cardiovascular system was proposed. The model involved: (1) a systematic analysis of the effect of various parameters on waveforms; (2) a patient-specific integration of multi-variable data measured at different sites; and (3) a mechanistic interpretation of clinical indices.

The model successfully integrated measured data for two participants (over the course of pregnancy and post-pregnancy) as inputs for the simulations. The model outputs compare favourably to physiological value ranges in the literature including: the pulsatility index, pulse wave velocity and expected increases in regional flow in the systemic arterial system. The model gave estimations of the previously unknown percentage of cardiac output which the utero-ovarian communicating artery supplies to the uterus, with an estimated 0.6 l/min for participant A and 0.57 l/min for participant B. The model could be used as a basis for future research, with a view to providing an early detection tool for the development of pathologies during pregnancy. Although this would require the study of many more participants, the model has been shown to give physiologically representative haemodynamic quantities. It is the first such model of pregnancy that can capture the various wave propagation phenomena, which are important for measuring arterial stiffness, and the first to estimate volumetric flow rate via the utero-ovarian communicating artery.

## Electronic supplementary material

Below is the link to the electronic supplementary material.

Supplementary material 1 (xlsx 48 KB)

Supplementary material 2 (xlsx 43 KB)
